# Supplementary material for: Computed tomography evaluation of risk factors for an undesirable buccal split during sagittal split ramus osteotomy
Source: PLoS One. 2023 Mar 2;18(3):e0279850. doi: 10.1371/journal.pone.0279850 (PMC9980734; doi:10.1371/journal.pone.0279850)
Supplement: Attachment — Submitted filename: Responses_to_Reviewers.docx [file pone.0279850.s002.docx]

December 13, 2022

**Dr. Tanay Chaubal**

Academic Editor

*PLOS ONE*

RE: Resubmission of manuscript “**Computed tomography evaluation of risk factors for an undesirable buccal split during sagittal split ramus osteotomy”** by Yasuyuki Fujii et al. (ID: PONE-D-22-09899R1)

Dear Dr. Chaubal,

We would like to submit a revised version of our above manuscript. We are most grateful to you and the reviewers for the helpful comments and insightful suggestions, which have helped us to substantially improve our manuscript. Please find attached our revised manuscript and our point-by-point responses to the reviewers’ comments.

We hope that the revised version of our manuscript is now suitable for publication in *PLOS ONE*. We look forward to hearing from you at your earliest convenience.

Sincerely yours,

**Yasuyuki Fujii, D.D.S., Ph.D.**

Department of Oral & Maxillofacial Surgery

Tokyo Medical University Hospital

6-7-1 Nishishinjuku, Shinjuku-ku, Tokyo 160-0023, Japan

Tel.: +81-3-3342-6111 (ext. 62724)

Fax: +81-3-3342-1723

E-mail: yfujii@tokyo-med.ac.jp

**Point-by-point responses to the Reviewers’ comments**

We wish to express our appreciation to the reviewers for their insightful comments, which have helped us to substantially improve our manuscript.

**Responses to Reviewer 3**

**Comment #1**

*Could you define the bad split in the introduction section?*

Response:

Thank you very much for this comment. We have revised the text in accordance with the comment (Introduction: p3, lines 44-45).

**Comment #2**

*You have divided the patients into two groups, control and bad split, although they underwent the same procedures/split technique. Bad split is considered as a complication rather than a technique. I suggest renaming the groups such as correct/good split and bad split instead of control and bad split to be less confusing for the readers.*

Response:

Thank you very much for this comment. To avoid confusion, we changed “control” to “good split” throughout the manuscript.

**Comment #3**

*9 Surgeons ran the surgery. These numbers of surgeons for 27 cases are too many and can increase the variability that can dramatically reduce your statistical power and impact the measurement and statistical data analysis, despite the presence of one supervisor. This point could be considered as a study limitation.*

Response:

We greatly appreciate and agree with this comment. In accordance with the comment, we have added this point as a limitation of the study (Discussion: p16, lines 273–275).

**Comment #4**

*Could you mention the age of the patients in the methodology section?*

Response:

Thank you very much for this comment. We have revised the text in accordance with the comment (Materials and Methods: p4, lines 69-71).

**Comment #5**

*You have mentioned that two surgeons measured the data. Is it for reliability or they shared the CT images to process them and collect data? If it was for interexaminer reliability, what is the correlation between the two measurements (surgeon 1 and 2)?*

Response:

We greatly appreciate and agree with this comment. Two surgeons performed the measurements to examine interexaminer reliability. Interexaminer reliability was quantified using intraclass correlation coefficients (ICCs). The ICC values for the measurements of the CT images were from 0.966 to 0.998. The ICC value for the measurements of bone quality analysis in Fig. 4 was 0.972. These results indicated excellent reliability. We have revised the text accordingly, and added this point to the manuscript (Materials and Methods: p9-10, lines 165-167, Results: p11, lines 186–187, 193-194).
